# The Role of Civic Engagement for Men’s Health and Well Being in Norway—A Contribution to Public Health

**DOI:** 10.3390/ijerph110606375

**Published:** 2014-06-18

**Authors:** Ursula S. Goth, Erik Småland

**Affiliations:** 1 Faculty of Education and International Studies, Oslo and Akershus University College, 0130 Oslo, Norway; 2 Faculty of Health Sciences, Buskerud and Vestfold University College, 3603 Kongsberg, Norway; 3 Faculty of Humanities, University of Bergen, 5020 Bergen, Norway; E-Mail: erik.smaaland@ra.no

**Keywords:** volunteer, social determinants of health, civic engagement, cultural heritage, elderly, health outcomes

## Abstract

*Objectives*: Using the example of older men volunteering on teams that restore historic ships, this article examines the effects of volunteering on the well-being of older adults. We consider particularly how volunteering impacts levels of social engagement and explore how the men’s reminiscences as they bond with their fellows in highly skilled work helps integrate their life experiences. *Methods*: Data are based on 14 in-depth interviews with volunteers working on historic vessels in Norway. Self-rated health, functional dependency, and well-being measures were collected using semi-structured questionnaire. *Results*: Volunteering in a context of skilled, group-bonded, culturally prestigious activity adds considerably to social capital among elderly men in Norway. Respondents explain their involvement in terms of prior relationships and current social benefits. They spoke of the value of maintaining past personal connections to a particular ship, shipping company, or local community. These were reinforced by current social benefits, such as the experience of companionship, unity, and the feeling of making an important contribution to the society. The group dynamics and strong collective aspect of these voluntary associations maintains internal cohesion, and members only leave when forced by increasing age, poor health, or insufficient financial resources. *Conclusions*: This article illuminates a case study of gender-specific engagement of older adults in volunteer roles returning high benefits both to participants and society, and adds knowledge to public-health programs and policies in the volunteer- and cultural-heritage sector.

## 1. Introduction

Positive health outcomes linked to volunteering may offer a promising avenue for increasing longevity, especially among older adults, who face increasing risks of poor health often compounded by social isolation [1]. In Norway there is a rising awareness about the need to involve adults in the community, and especially older men. Creating opportunities for participation in civic engagement in later life can have a significant impact on the social aspects of live and health in general. The present study examines an important, but not widely appreciated, dimension by which health and health-equity can be promoted: via policies that facilitate and nurture—without directing—community participation in group-based volunteer efforts. Such policies are particularly important if geared toward those facing greater social inequality.

With this focus, this article seeks to contribute to the understanding of tailored, voluntary activity a specific age group that finds itself often without an attracting activity or engagement possibility. In this article we focus on mature men and their experiences volunteering to restore or operate historic ships in Norway.

### 1.1. Volunteer Work and Activities at a Societal Level

The term civic engagement is derived from civil society. These terms serve to contrast the space for social engagement, community, mutual recognition, and pursuit of interests that exists among individuals, and in contrast to the state and its power [2] or economic relations. Voluntary activities define civic engagement. Structures for civic engagement might range from local *ad-hoc* groups to permanent organizations with local, regional, national, and international branches. These structures serve to allow individual social networks to extend beyond neighbors, family, and other inherited links, to include people who share one’s personal views and interests nationwide or beyond.

A key aspect of social isolation among the elderly relates to loss of a spouse, one of the biggest losses a person can experience. There are differences in how older men and women perceive this [3]. Men tend to socialize outside the home, in contrast to women who tend to maintain social contact with friends and family at home. Here women meet to chat and vent emotions, while men tend to seek out groups with a shared activity as focus [3].

Volunteer work is an essential aspect of civic engagement, and researchers highlight its essential aspects as being non-commercial and autonomously chosen [4,5]. While not all forms of civil engagement might be seen as unambiguously socially positive—e.g., vigilante groups, or mobilizations against immigrants—most are skewed in pro-social directions. Seafaring has from time immemorial required the tightly integrated labor of isolated groups of males together facing enormous risks, usually for the good of their communities, and demanding high levels of bonding, coordination, and sacrifice over long periods, so it is a *locus classicus* of many of these dimensions of the pro-social. Especially in the Norwegian context, with its maritime traditions so culturally fundamental, working together in a voluntary association aiming to restore or maintain historic ships is an example of civic engagement that serves broad interests of cultural stability, collective identity [6], and social cohesion [7]. An aspect of this self-reinforcing virtuous circle is how civic engagement promotes the health of volunteers by increasing their social capital [8], which is linked in turn to broad societal measures of communal health [9] as shown in epidemiological studies by Berkman and Glass [10]. The linkage between high social capital and rich communal ties on the one hand and individual health on the other can be seen, for instance, in how life expectancy is greatest when these come together. Intriguingly, community size does not appear to be relevant variable [8].

Restoration of historic ships by volunteers has a long tradition in Norway [6,11]. Most historic ships in Norway are privately owned, however their restoration is often funded by the state, but only insofar as covering cost of equipment, parts, materials, and specially-commissioned work, with no reimbursement for labor [6]. Despite public funding, the work of restoring and maintaining these ships is directed and handled by crews of volunteers [6]. With its maritime traditions historically central to Norwegian identity, the preservation of old ships is culturally prestigious. Volunteers in this area both enjoy the status of doing work with recognized meaning and represent a significant resource in Norwegian society [6]. Volunteers are mostly men between ages 17 and 85, with some two-thirds in their 50s and 60s [6].

One dimension of a larger trend toward higher individualism in Western societies is suggested by research that indicates a rising commitment and growth in non-organized volunteering, focused on the personal rewards of altruistic service but without the requirement of formal memberships [12]. Whatever its structure or motivation, volunteers’ efforts on the ships receiving state funding in Norway was estimated for 2009 to have a value of approximately 5.5 million euros in unpaid work [6].

Social benefits of volunteering redound to the volunteers themselves, and in particular show a positive health impact. The authors further conclude that socially-inclusive volunteering yields health benefits, with especially positive effects on older people.

Social inclusion has been widely recognized as a key social determinant of health. As defined by the World Health Organization, the Social Determinants of Health (SDOH) are the conditions in which people are born, grow, live, work, and age; included in this conception, but extending well beyond it, is the local health system [7]. These circumstances are shaped by the distribution of money, power, and resources at global, national, and local levels, which again are influenced by policy choices [7]. In 2008 the World Health Report showed that health and health equity are a function of determinants that are broadly categorizable as social, cultural, and material. Every aspect of government and economy has the potential to affect these outcomes. In a specific situation, of course it might be difficult isolate, for instance, social and cultural determinants.

### 1.2. Volunteering: Impact on an Individual Level

Each volunteer experiences volunteering differently, but shifting overall patterns can be seen. Today there is a growth in individual, spontaneous, non-organized participation among younger adults in Norway, suggesting a changing picture of civic engagement, away from more organized and structured forms [13]. In this study we understand the latter as traditions—group-specific frames of understanding and structures of meaning.

As participation as is not only seen to strengthen ties to the local community, it is also seen as a potential pathway for social capital to influence health outcomes [8]. Improving health and well being require empowering individuals by community participation and social cohesion [7]. Recognizing this impact has led to a focus on SDOHs in health policies internationally [7].

At the same time more and more elderlies are keeping up their activities in traditional organized volunteering [13]. According to the continuity theory, adults show inertia in persisting with previous and present activities and mental structures. By adapting strategies to sustain previous activities the individual experiences the feeling of continuity [14]. Gender and age are significant parameters of choices of leisure-time activities including volunteer work. International literature tells us that most volunteers come from wealthier, more educated sections of society, are female, and married with children [15]. Age as well as gender are significant parameters of how leisure time is used. Motivations for volunteering change during the life cycle. Research showed that volunteers with children living at home are less likely to volunteer, and spend less hours in volunteer work [16]. Divorced males are more likely to volunteer than divorced females, but older widows tend to increase their volunteering activities [16]. There is equal involvement in volunteer work between genders in older age, but woman are more socially active earlier in life [17]. A local study shows that male volunteers over the age of 67 are the most active with regard to volunteer efforts [18]. A volunteer activity that particularly attracts older men, and those skewed toward “blue-collar” rather than “white-collar” backgrounds, is a particularly interesting case study. In a previous study we found that volunteering within ship preservation attracts this group [6]. We go on now to explore deeper the reason for this attraction, examining the reasons, motives, rewards, and broader social and health impacts of this participation. In Norway research shows that younger women participate more than younger men but that older men participate more than older women [13].

Living in fast-paced, continually technologically destabilized modern culture is often experienced as volatile. The experienced reality fills up with the new and not-yet-categorized while the known and appreciated is fading. This process even involves cultural categories and concepts. The feeling of loss generated can mobilize volunteers to organize, save and restore key objects that incarnate important meanings and experiences of the past. Restoring these objects together and reliving memories prevents the feeling of loss and uncertainty, and simultaneously generates new sources of social capital. These themes are clearly displayed in the interviews, where volunteers refer to personal experiences and memories as they speak about what motivates and rewards their volunteer work.

As volunteers contribute to society, they also earn social appreciation and respect. Findings from a number of studies show a strong relationship between volunteering and health [19], as well as self-worth. Both social inclusion and the gaining social capital might here be seen as reward for volunteer work. A model for this is offered by Social Integration Theory [20], which suggests how multiple social roles provide meaning and purpose in life and additionally promote social support and interaction.

Social activities alone encourage feeling good about oneself [21]. Volunteering can support not just hedonic feelings of well-being [22], but personal development and growth, and a sense of finding meaning in one’s life. The positive impact not only directly benefits individuals but also those close to them [15,23].

Empowerment of elderlies as defined by WHO is understood in relation to the social discrimination older persons experience by being excluded from decision-making by age or disability [24]. Barriers to empowerment lie ingrained in current Western cultural norms, abetted by fast technological change and a valorization of novelty and youth. Additionally elderly people often face problems to adapt to the changes arising simply from old age. Frequent consequences are experience of powerlessness, helplessness, low self-esteem, and low self-efficacy [25]. To prevent elderlies from becoming passive and facing the risks to health social withdrawal entails, contact with the community needs to be supported.

Feeling both good about one’s situation in life and about oneself by empowerment, enforces a greater sense of satisfaction; namely to contribute to society [15]. This sense of “to matter” or “to make a difference” are significant for everyone. It is easy to overlook health implications, yet such meaning is implicit in the definition of “health as a state of complete physical and mental and social well-being and not merely the absence of disease or infirmity” [26].

International research demonstrates that volunteering leads to better health and that older volunteers are those most likely to receive physical- and mental-health benefits from their volunteer activities [15,27,28,29]. This effect might be connected to “culture” through the concept of reminiscence.

In one review, volunteering as a public-health promotion intervention, was found to improve physical and mental outcome variables [30]. Another longitudinal study, originally including over 10,000 informants, showed that the consistency of volunteering over time is positively related to both well-being and self-reported health [15]. Piliavin and Siegel’s study showed further that less-integrated volunteers benefit the most. Other studies propose that older adult volunteers may enjoy good health and longevity because being useful to others instills a sense of being needed and valued [31]. Here Morrow-Howell *et al*. conclude in their study that there is a correlation between hours of volunteering and a higher level of well-being, independent of social integration, race, or gender [28].

Recently published research shows that the feeling of purpose in life may extend ones lifespan [32]. These findings underscore that the positive effects of volunteering for elderlies is more than an effect of selection bias, presuming that healthier elders are likelier to volunteer in the first place. An ideal of “active aging” especially warrants extension to a wider proportion of vulnerable elderlies characterized by low activity and social withdrawal.

## 2. Methods

### 2.1. Definitions

*Volunteering* as used in this article is understood as an organized, private, self-governed activity without a predominantly commercial purpose or expectation of personal profit. This is leading to the assumption that non-profit organizations promote common good and that volunteer’s work is performed without the expectation of personal profit [4].

*Empowerment* is in this article seen as an approach to increase the capacity to act both of individuals and groups of people, and entire communities, through a combination of top-down and bottom-up processes [33]. It is furthermore seen as an approach, mounting in a community capacity-building process.

### 2.2. Data

Our data are based on 14 in-depth, semi-structured interviews, from randomly selected volunteers working on ships receiving funds from the Directorate of Heritage for the year 2009 [6]. Representing the field of volunteering on historic vessels the 14 informants were male, age 18 to 74, representing an age distribution common in the field of volunteer work on historic ships [6]. Two were in the age group 18–30, one in the group 31–50, ten in the group 51–70, and one informant in the group 71 and more. This represents the overall age distribution in the field of volunteer ship preservation in Norway [6]. All except one of the interviews took place on-board the individual volunteer’s vessel, were based on a semi-structured interview guide, and varied between 60 to 90 min and the informed consent was signed before the actual interview took place.

### 2.3. The Data Collection, Analysis and Ethical Approvement

These interviewees were each participants with one of the 82 Norwegian volunteer organizations receiving state funding for preserving and maintaining a total of 90 historical ships in 2009 [6]. Data saturation was achieved by the 13th interview [34].

After each interview the authors coded, clustered, and grouped and thereafter analyzed the gained data on the basis of grounded theory [35]. The analysis started with open coding in order to capture important concepts and themes and to illustrate central statements. Deductive methods were used to assess variation in the material. Next we identified key themes that emerged across all groups and interviews, as well as unique issues mentioned only by a single respondent, all based on reflection on the material by authors [34]. Based on the analyzed data, the following categories emerged: (1) Relationship to the individual ships or site; (2) Expectations and experiences of companionship and (3) Experience of empowerment.

To control for bias and to assure consistency both authors (Ursula S. Goth and Erik Småland) validated the data individually, thereafter comparing the results [6,34]. All interviews were recorded on tape and transcribed thereafter. The Regional Committee for Medical and Health Research Ethics (Norway) approved this study (reference number 2010/173a), though the authors accept responsibility for all limitations.

### 2.4. Limitations of the Study

The interviewer (Erik Småland) has experience as a volunteer and is employed by the funding body, a factor that might unconsciously bias reliability and validity. To counteract that risk, the co-author (Ursula S. Goth) analyzed all data after transcription and before acknowledging the interpretation of Erik Småland. The hermeneutic impact that the interviewer might have on interviewees could not be adjusted for. Additionally, a recall bias by the volunteer must be accounted for, because the interviewed volunteers relied solely on their memory. Furthermore the small number of informants included in this study limits the validity of the results and can only indicate the motives and impact for mature men already engaged in volunteer work in Norway.

## 3. Results and Discussion

To enhance the contents of the study, the four major categories the emerged from the analysis are consecutively presented and illustrated with empirical data [36].

### 3.1. Relationship to the Individual Ships or Site

“I grew up with boats—ferry boats I mean. When I was a boy we went on the dock every time the ferry came and took the rope and… So I have a tremendous connection with these boats.” *(Volunteer 1, male, 53 years of age)*.

The volunteers interviewed showed a distinctive, often personal, relationship to the particular ship on which they volunteered, or to the ship’s original company. A background in maritime-related jobs was frequently mentioned. For many older respondents, their volunteer work was a form of reminiscence, a framework on which they could organize, remember, and retell personal stories, allowing them to live vicariously in “the old days”. The boats moreover, as immediately present objects, gave tangible and public reality to their personal experience and a shared heritage [11]. As publicly prized objects—the government was after all funding their restoration—the boats linked these personal experiences to a shared heritage and represented a means of projecting their memory into the future.

“We grew up there—so we have been here since childhood, where we used to travel by boat to the town!” *(Volunteer 9, male, 71 years of age)*.

Volunteers often emphasized their personal interest in a particular ship or the company that had operated it, perhaps through past employment or other connections with their own lives. These connections were varied and often woven into the individual’s life narratives [6]. The particular vessel was experienced as a “touchable part” of history, helping bridge both the individual’s own, as well as the larger social, present and past. Those inter-temporal connections, moreover, were validated by being part of a shared recognition, strengthened by the fellowship keenly felt with the other volunteers.

“…old boats, are like pieces of jewelry. I cannot explain it. I am so fortunate to be able to participate. Yes, it involves lots of work, but we have it wonderful, are a nice crowd and experience nice day trips.” *(Volunteer 6, man 53 years of age)*.

Volunteers showed a highly special interest and personal relationship for one particular ship or the ship’s original company. Based on our data we see the importance of local history in attracting many of the elderly volunteers, as well as the validation that comes from meeting and working with others who share their interests.

Within the Continuity Theory of normal aging [14] we see that adults preserve and maintain mental structures by using past experiences of themselves and their social world. This perspective clearly applied to our data, as volunteering proved closely related to personal ties and respondents’ personal sense of history. Moreover, these individuals’ memory was shown to be a vital constitutive element of identity-formation—unlike professional historical narratives, it is personal and invested with emotive power [37]. A previous Norwegian study likewise found that the most frequent motivating factors among volunteers in the heritage sector care closely related to personal experience [38], bolstered with ideas about the intrinsic value of the labour itself [39].

### 3.2. Expectations and Experiences of Companionship

“…I was attracted to the atmosphere. One thing are vessels and heritage protection and another is the environment on board. Here I met exciting personalities. And maybe—silly to say—a masculine environment. A somehow rough environment.” *(Volunteer 7, male, 58 years of age)*.

Previous research shows the main group of volunteers in the field of ship preservation are men over the age of 50 with the desire to join a fellowship and at the same time to support a cause that they believe to be important [6].

But our data also indicate that deep knowledge and skills many of the volunteers brought to this work—skills and experience historically associated with working in groups of males—was also of key importance. A similar finding is seen in a study by Mjelde-Mossey and Chi [40]. We therefore assume that the gendered character of this volunteer work was seen as a positive factor. That volunteers recruit primarily from their social networks and within their own age groups leads to the assumption that work on historic ships becomes something that both binds and animates the social network of a growing segment of older men [6].

“…using historical techniques (methods), and to meet like people similar to me. The social aspect was the most important.” *(Volunteer 5, male, 66 years of age)*.

Our data shows the strongest motivation for participation is the social aspect and having a good time. These expressions of unity and companionship were emphasized in nearly every interview. As those activities present an arena for social cohesion the vessel is recognized as a meeting point, a place where they might feel needed in their effort to serve their community along with their peers.

“…well just to have fun. I like it here very much, and therefore I continued to come.” *(Volunteer 13, male, 18 years of age)*.

“I’ve got good friends there (at the vessel), you might say. So this has meant a lot to me personally, it really has.” (*Volunteer 9, male, 71 years of age)*.

Volunteering was described as a function of social interaction between the individual and his social surroundings. By volunteering, new links are created connecting people outside of their immediate circles and providing ties to friends and neighbours at a point in life when ties to workplace and family are reduced or even disappearing.

### 3.3. Self-rated Health, Well-Being, and Living Situation

As adults are ageing, it is likely that they retire from work, with friends and partners increasingly die off. Their children have left and live far away [3]. Engagement in volunteer activities can strengthen social ties and protect from isolation. During interviews, all volunteers rated their own health as good—even in a case where the respondent was diagnosed with cancer, self-rated health and well-being was experienced as good.

As previous research shows, the link between volunteering and well-being is connected to the experience of “mattering” outside ourselves [15]. This feeling of contributing to society supports a feeling of satisfaction greater than the merely hedonic [15]. In this study, this well-being is a reservoir individuals can draw on when experiencing disease or ill health. Such connection to others having a positive health impact is statistically supported in earlier findings [15,32], as well as being supported by Casiday, Kinsman, Fisher and Bambra [41], who found that volunteering can increase volunteers’ longevity, improve mental health, keep them fitter and enable them to cope better with illness when it occurs. Other research found that volunteers were at lower risk for mortality, especially those who volunteered more regularly and frequently [1].

The social capital volunteering builds is robustly related to subjective experience of well-being, happiness, and life satisfaction, both directly and through its impact on health [42].

“…to be able to experience something like… something that would end up in a two-month long boat trip… us being such an attention all along the coast… I could never have dreamed of that.” *(Volunteer 9, male, 71 years of age).*

The feeling of being needed, respected, and appreciated girds the experience of positive self-worth. This feeling of self-worth is created by volunteering was a subtext during the interviews but surprisingly was not often directly communicated.

“My health is good, although I have cancer. So I’m ok.” (*Volunteer 12, male, 66 years of age)*.

Self-rated health is an indicator of physical well-being. Studies indicate that areas with higher volunteer rates have lower mortality rates and less incidence of heart disease. This indicates that volunteers are more likely to experience positive health benefits that those who do not [32,43]. Even when controlled for variables such as age, health, and gender research has found that volunteering is most likely prolonging the lifespan of the individual [44].

“My health is good… Maybe I feel a bit responsible for the folks at home. I should also be home sometimes.” *(Volunteer 10, male, 18 years of age)*.

Self-rated health was experienced as non-important by one of the young informants as a hindrance to volunteering, but in contrast to elder volunteers, responsibilities at home and to family was mentioned as a competing factor. Older respondents tended to express the alternative to volunteering in terms of “sitting at home”, which suggests possible resignation to inactivity—while the young expressed the alternative as “going around at home”, suggesting rather impatience and boredom.

“It’s great to have something to do when I am not working. Or else I will just be going around at home.” *(Volunteer 13, male, age 18)*.

“The social part is of importance… since I am retired now—you know—I am just sitting at home.” *(Volunteer 2, male, age 70).*

The diverse living situation of those two volunteers demonstrate a different approach to volunteer activities.

### 3.4. Experience of Empowerment

“I started a little careful… what I expected was related to historical technique but also to meet other like-minded people. The social was the most important thing… Now I meet the people who are on the line with me right away… I wish to continue. It’s clear that when you retire, it’s important to have something that is so meaningful.” *(Volunteer 5, male, 66 of age)*.

Here the experience of empowerment was seen as a process in interactive stages in which the individual’s own awareness of the value of participation expands and leads to developing new relationships, skills, and forms of knowledge, and enhancing self-esteem.

Volunteering is not only an advantage for the community, but has also a positive impact for the individual volunteer [15,31,32]. Elderlies and especially older men are a group exposed to processes leading to marginalization and loneliness. This might be counteracted by participation in activities enforcing one’s own social network and hence social capital [33].

## 4. Conclusions

The results of our study indicated four major reasons for participation: the feeling of unity and companionship, a relationship to the individual ship, a more general relationship to the region or its nautical environment, and the experience of empowerment. All volunteers included in this study highlighted these as motivating factors, but differed in sequence and weighting. A strong connection between volunteering and the concept of salutogenesis—processes that foster health—is clearly shown. These results emphasize that volunteering contributes to social capital among those directly networked. In the domain of national heritage, such activities and the objects preserved encourage reflection on collective cultural history, enhancing social cohesion and communal identity in the face of the destabilizations wrought by modernity and post-modernity. Indeed, the feeling of loss that something experienced as important is disappearing (among objects, traditions, and concepts) that might create feelings of loss of identity here provokes an individually and civically salutogenic response. In this case, these diverse benefits are made possible by the initial investment of government grants and project funding.

Projects such as volunteering on historic ships attract primarily skilled men in higher age groups. As men in this category are broadly lacking available volunteer opportunities, favoring such projects can usefully be part of public-health policies.

Although the 14 interviews shows individual examples of higher rating of well-being and health benefits from volunteering on historic ships, we cannot provide statistical proof of health impact given the research design. But evidence from other studies shows that volunteers with similar characteristics display higher self-perceived health and longer lifespan. In this context we conclude that tailored activities targeting certain gender and age groups, especially those with currently fewer volunteer opportunities—and hence missed opportunities for making a unique social contribution—will have a positive effect on basic markers of health.

Further research is needed to outline gender and age-specific preferences to maximize the opportunities and the social and health benefits of volunteering, as an aspect of optimizing public-health policies in the future.
